# The Association between the Five-Minute Apgar Score and Neurodevelopmental Outcomes among Children Aged 8−66 Months in Australia

**DOI:** 10.3390/ijerph18126450

**Published:** 2021-06-15

**Authors:** Tahir Ahmed Hassen, Catherine Chojenta, Nicholas Egan, Deborah Loxton

**Affiliations:** Centre For Women’s Health Research, School of Medicine and Public Health, College of Health, Medicine and Wellbeing, The University of Newcastle, Callaghan, NSW 2308, Australia; catherine.chojenta@newcastle.edu.au (C.C.); nick.egan@newcastle.edu.au (N.E.); deborah.loxton@newcastle.edu.au (D.L.)

**Keywords:** Apgar score, neurodevelopment, children, Australia

## Abstract

This study aimed to evaluate the association of the five-minute Apgar score and neurodevelopmental outcomes in children by taking the entire range of Apgar scores into account. Data from the Australian Longitudinal Study of Women’s Health (ALSWH) and Mothers and their Children’s Health (MatCH) study were linked with Australian state-based Perinatal Data Collections (PDCs) for 809 children aged 8−66 months old. Generalized estimating equations were used to model the association between the five-minute Apgar scores and neurodevelopmental outcomes, using STATA software V.15. Of the 809 children, 614 (75.3%) had a five-minute Apgar score of 9, and 130 (16.1%) had an Apgar score of 10. Approximately 1.9% and 6.2% had Apgar scores of 0−6 and 7−8, respectively. Sixty-nine (8.5%) of children had a neurodevelopmental delay. Children with an Apgar score of 0−6 (AOR = 5.7; 95% CI: 1.2, 27.8) and 7−8 (AOR = 4.1; 95% CI: 1.2, 14.1) had greater odds of gross-motor neurodevelopment delay compared to children with an Apgar score of 10. Further, when continuously modelled, the five-minute Apgar score was inversely associated with neurodevelopmental delay (AOR = 0.75; 95% CI: 0.60, 0.93). Five-minute Apgar score was independently and inversely associated with a neurodevelopmental delay, and the risks were higher even within an Apgar score of 7−8. Hence, the Apgar score may need to be taken into account when evaluating neurodevelopmental outcomes in children.

## 1. Introduction

Developed by Virginia Apgar in 1952, the Apgar score has been used as a rapid assessment of the clinical condition of newborns based on physiological functions, such as respiration, heart rate, skin colour, muscle tone, and reflex irritability [1]. Commonly, the Apgar score is measured at one minute and five minutes after birth and rated from zero to two points for each component, giving a total score that ranges from one to ten, where a higher score indicates better health and a greater chance of survival. Compared with the one-minute Apgar score, the five-minute Apgar score is a better predictor of survival [2].

The Apgar score was originally intended to assess the condition of newborns immediately after birth and to measure the response to resuscitations [3]. However, the Apgar score, particularly the five-minute Apgar score, is often used in outcome studies, as it provides useful clinical information about the fetal-to-neonatal transition [4,5,6], although some professional associations, such as the American Academy of Pediatrics and the American College of Obstetricians and Gynecologists, encourage the use of the Apgar score only for its original intent [7].

Generally, an Apgar score of seven or more at five minutes after birth indicates a newborn is adapting well to the environment, while a score of less than seven indicates complications [8]. A low Apgar score, commonly defined as a score of less than seven, has been associated with an increased risk of neonatal morbidity, mortality [9,10], and neurodevelopmental outcomes, such as motor, language, and educational outcomes [11,12,13].

Conventionally, only severely compromised and low Apgar scores have been regarded as predictive of maladaptive development and morbidity [14,15]. However, emerging evidence indicates that the risk of poor neonatal outcomes [16] and adverse neurodevelopmental outcomes increases as Apgar scores decrease [4,6,17], highlighting the need to consider the Apgar score as a nuanced continuous measure rather than as a blunt, dichotomous construct.

When evaluating the risk associated with five-minute Apgar scores, taking the entire range into account is very important, as the vast majority of births fall into what has conventionally been regarded as the normal range, which is assumed to be associated with no/minimal risk [18]. For example, in Australia, in 2018, about 98% of live-born, term babies had an Apgar score of seven or more [8]. In another way, if only children with Apgar scores of below normal range are considered, only two percent of livebirths will be included, and the vast majority of births will be grouped together, implicating the presence of similar risks between them, which might not be true. Despite this, however, only limited studies have attempted to account for this large segment of the population when analyzing the risks associated with five-minute Apgar scores [5]. Therefore, this study aimed to evaluate the association of the five-minute Apgar score and neurodevelopmental outcomes in children by taking the entire range of Apgar scores into account.

## 2. Materials and Methods

### 2.1. Data Sources and Sample

Data from the Australian Longitudinal Study of Women’s Health (ALSWH) and the Mothers and their Children’s Health study (MatCH) were linked to data from the Australian state-based Perinatal Data Collections (PDCs). The ALSWH is a longitudinal, population-based survey that has been conducted since 1996 with three different cohorts: women born 1921–1926, 1946–1951, and 1973–1978 [19]. Participants were randomly selected from the Medicare database (the Australian universal health insurance system), and women from rural and remote areas were sampled at twice the rate of women in urban areas to provide sufficient representation.

The MatCH study is a sub-study of the ALSWH that aimed to investigate how maternal and family characteristics impact the health and development of the next generation. Women in the 1973–1978 cohort of the ALSWH who had reported at least one live birth were invited to complete additional surveys in 2016, either online or on paper, about their biological children under 13 years [20].

The PDCs are Australian state-based data collections for pregnancies and births that include both live births and stillbirths [8]. The current study is based on the children (aged 8−66 months) of women in the 1973–1978 cohort who had completed an online survey for the MatCH study (n = 809). We restricted the sample to children of mothers who completed the online version of the survey, because the paper version of the survey did not measure neurodevelopmental outcomes. All the three data sources (ALSWH, MatCH, and PDC) were linked and used in this analysis.

### 2.2. Variables and Measurements

#### 2.2.1. Outcome Variables

The outcome variables were neurodevelopmental outcomes in children and were measured using the parent-completed Ages and Stages Questionnaire version 3.0 (ASQ-3) [21] included in the MatCH study. The ASQ is a widely used, age-appropriate neurodevelopmental questionnaire based on the five domains of child development: communication, gross motor, fine motor, problem-solving, and personal-social [21,22]. The questionnaire is applicable for 1−66 months old children and consists of 30 developmental items. The parents rated the ability of the child to perform certain tasks as “yes”, “sometimes”, or “not yet”. Each domain score was obtained by the sum of the items, compared with established cut-off screening points, and was considered abnormal if the score was 2 standard deviations below the mean [21,23]. Neurodevelopmental outcomes were expressed as communication and gross motor delay in this study.

#### 2.2.2. Exposures

The five-minute Apgar score was the main exposure variable in this study, and it was obtained from the PDCs. The score was used both as a categorical and a continuous variable.

#### 2.2.3. Covariates

We identified potential covariates based on a review of the available literature. The following covariates were included in the study: mother’s country of birth, mother’s area of residence, mother’s age at birth, marital status, smoking during pregnancy, gestational hypertension, gestational diabetes, mode of birth, gestational age at birth, birth weight, and age of the child, sex of the child, child medical problems, and the child’s average screen time per day.

### 2.3. Data Analysis

Data cleaning and management were performed and followed by a descriptive analysis of main exposure variable (five-minute Apgar score). The Apgar score was presented both as a continuous variable and a categorical variable. The mean and standard deviation (SD) were presented for the continuous Apgar score. Further, maximum efforts have been made to present the entire range of Apgar score (0−10) as individual categories. However, due to low frequencies of lower Apgar scores, some individual scores were collapsed and are presented as scores of 0−6, 7−8, 9, and 10. We then summarized maternal and child characteristics according to the Apgar score categories.

The neurodevelopmental outcome variable data were coded as per the ASQ-3 manual (“not yet” = 0 points, “sometimes” = 5 points, “yes” = 10 points) [21]. The missing items were also handled according to the ASQ-3 manual, that is, when less than or equal to two items were missing, the items were imputed with the average score, which was obtained by dividing the total domain score by the number of items answered in the specific domain. Adjusted total domain scores were then computed by adding the average scores that were imputed for the missing items into the scores of the items with nothing missing (the answered items) [21].

In order to investigate the association between five-minute Apgar scores and neurodevelopmental outcomes, the items in each domain were added together, giving the total score for each domain. When the total score was two standard deviation below the mean, children were categorized as having a neurodevelopmental delay for the particular domain.

Generalized estimating equations (GEE) [24] models were fitted to examine the association between five-minute Apgar scores and neurodevelopmental outcomes, with children nested within mothers. GEE models are a flexible, regression-based approach for dealing with clustered or correlated data [24,25].

Two models were constructed. The first model included five-minute Apgar scores that were grouped into four categories and were adjusted for the potential covariates to evaluate whether there was variability within the reassuring range (7–10). In the second model, the five-minute Apgar score was included as a continuous variable and was adjusted for a wide range of maternal and child characteristics.

Adjusted odds ratios with 95% confidence intervals (CI) were used as estimates of the association between five-minute Apgar scores and neurodevelopmental outcomes. All analyses were performed in Stata 15.0 (StataCorp LLC, College Station, United States [26]. Table cells with small counts (less than five) were not published to maintain confidentiality as per the Data Custodian’s policy.

## 3. Results

Data for 809 children provided by 669 mothers were included in this analysis. The mean age of the children at time of completion of the survey was 42.4 months (SD = 15.6), and male children accounted for 54.0% of the sample. The mean five-minute Apgar score was 9 (SD = 0.8). As expected, the majority (75.9%) of children had an Apgar score of 9 followed by an Apgar score of 10 (16.1%). Approximately 1.9% had an Apgar score of 0−6, and 6.2% had an Apgar score of 7−8. The majority of children were born to mothers who were born in Australia (93.8%) and who were partnered (79.9%), and close to two-thirds of children were born through non-caesarean birth. Low Apgar scores were comparatively more common among males and children born through caesarean birth (Table 1).

**Figure 1:**
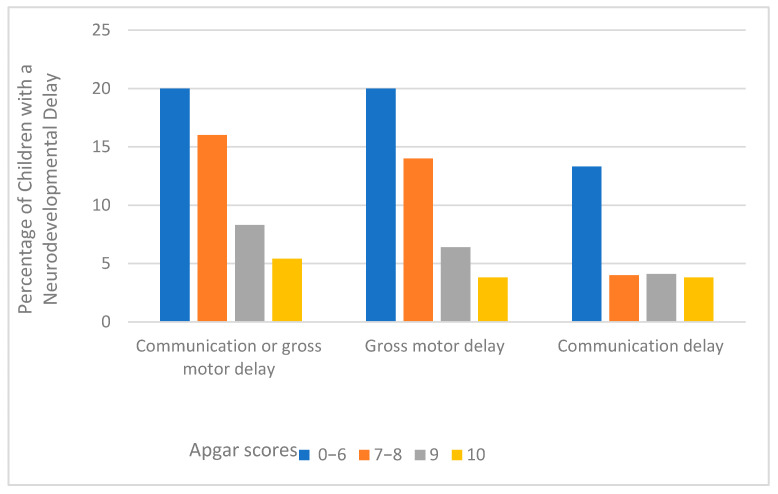
Percentage of neurodevelopmental delay by the five-minute Apgar scores among children aged 8–66 months.

The prevalence of any neurodevelopmental delay (communication or gross motor delay) in this sample was 8.5 %. About 7.1% and 4.2% of children had a gross motor delay and communication delay, respectively. Figure 1 presents the percentage of children with a neurodevelopmental delay according to the five-minute Apgar score categories. Overall, as the five-minute Apgar score increased, the proportion of children with neurodevelopmental delay decreased, although a significant amount of difference was not observed in the communication domains for the Apgar scores 7 and above.

After controlling for potential confounders, the odds of neurodevelopment delay in the gross motor domain were higher among children with an Apgar score of 0−6 (AOR = 5.7; 95% CI: 1.2, 27.8) and 7−8 (AOR = 4.1; 95% CI: 1.2, 14.1) compared to the children with an Apgar score of 10. Nevertheless, although the risks of having a neurodevelopment delay still appeared to be higher among children with an Apgar score of 0−6 and 7−8, compared to those with an Apgar score of 10, significant associations were not observed for the communication delay (Table 2).

When modelled as a continuous variable, the five-minute Apgar score showed a significant association with a neurodevelopmental delay in both communication and gross motor domains. That is, a one-unit increase in Apgar score reduced the odds of neurodevelopmental delay by 27% (AOR = 0.73; 95% CI: 0.55, 0.97) in communication and by 28% (AOR = 0.72; 95% CI: 0.57, 0.90) in gross motor domains. Similarly, a one-unit increase in Apgar score reduced the odds of neurodevelopmental delay in either communication or gross motor domains by 25% (AOR = 0.75; 95% CI: 0.60, 0.93) (Table S1).

## 4. Discussion

This study examined the association between five-minute Apgar score and neurodevelopmental outcomes among children aged 8−66 months. The findings of our study showed an association between the five-minute Apgar score and neurodevelopmental outcomes. That is, when modelled as a continuous variable, a unit increase in Apgar scores linearly decreases the risks of neurodevelopmental delay in both domains. The risk of neurodevelopmental delay in gross motor skills was found to be higher among children with 0−6 and 7−8 scores compared to children with an Apgar score of 10, indicating that the risks of neurodevelopmental delay are higher not only among children with an Apgar score of less than 7, as conventionally regarded, but also among children with a score of 7−8. Nevertheless, non-significant associations were observed between the five-minute Apgar score categories and communication delay.

Our finding is in line with large population-based studies conducted in Canada [4,6] that found a significant association between the five-minute Apgar score and developmental vulnerability in the physical health and wellbeing (gross motor) domain and non-significant associations between the five-minute Apgar score (across the entire range) and vulnerability in communication domain at age five, although the study tools are slightly different. Further, a recent population-based study from Sweden also showed an increased risk of poor neonatal outcomes among children with an Apgar score of 7, 8, and 9 compared with those with an Apgar score of 10 [15]. It is important to note, however, a significant association was not observed between an Apgar score of 9 and neurodevelopmental delays in the current study. This discrepancy could be attributed to different factors, such as the sample size, which might have impacted the associations that could have been observed if a large, population-based sample was considered in the current study.

These findings highlight the need to document the Apgar score as accurately as possible, because a small shift in the scores may misclassify a newborn and mislead the practitioners about the health and health-related outcomes of the newborn infant [4]. In addition, understanding the risk differences between different values of the Apgar scores would also provide a good reminder to avoid, whenever possible, dichotomizing the Apgar scores at a score of less than 7 and to adhere to consider an Apgar score of 10 as an optimal score. This is important because the scores do not carry similar risks even within the reassuring range. Thus, a gross categorization of the Apgar scores at a score of less than 7 may provide misleading information.

This study has notable strengths. Several important factors were accounted for when investigating the association between Apgar score and neurodevelopmental outcomes through data linkage approach. The study utilized a standardized and age-appropriate questionnaire to measure the neurodevelopmental outcomes and included children with different age ranges, enabling assessment of neurodevelopmental outcomes according to age groups.

Although a standardized questionnaire was used, data were not available for some domains of neurodevelopmental outcomes, such as fine-motor and problem-solving domains, warranting future research that addresses all domains of neurodevelopment. The sample size was also not large enough to consider and evaluate the entire value of the Apgar score as an individual score, particularly scores of less than 7. There might have been misclassification of newborns when Apgar scores were documented, due to inter-observer variability that often influences Apgar scores [27,28]. Finally, although many confounding factors that might influence the associations between five-minute Apgar score and neurodevelopmental outcomes were addressed in this study, still, there may be other factors (for example, the level of birth center and the level of resources available for neonatal resuscitation) that might affect the outcome of newborns with a low five-minute Apgar score, making the five-minute Apgar score of some limitation to predict neurodevelopmental outcome in children.

## 5. Conclusions

Our study indicated that, after accounting for perinatal and other important factors, the five-minute Apgar score was linearly and inversely associated with a neurodevelopmental delay in children. The study also demonstrated that the risks of neurodevelopmental delay, particularly gross-motor delay, were higher both among children with an Apgar score of 0−6 and 7−8 scores compared to children with an Apgar score of 10, indicating that not only children with conventionally low Apgar scores are at risk for adverse neurodevelopmental outcomes but also those with a lower-normal Apgar score. Thus, as a small degree of physiological abnormalities that are reflected in the Apgar score could impact the neurodevelopmental outcomes, the Apgar score should be documented as accurately as possible and may need to be taken into consideration during the assessment of neurodevelopmental outcomes in children. Further, early interventions might be required for children with lower Apgar scores to reduce the risk of neurodevelopmental delay.

## Figures and Tables

**Table 1:** Maternal and child characteristic according to the five-minute Apgar scores among children aged 8−66 months.

Factors	Five-Minute Apgar Scores									
	Total		0–6		7–8		9		10	
	No	%	No	%	No	%	No	%	No	%
Mother’s country of birth										
Australia	754	93.8	14	1.8	49	6.5	564	74.8	127	16.8
Not-Australia	50	6.2	n.p	n.p	n.p	n.p	45	90.0	n.p	n.p
Mother’s area of residence										
Major cities	557	70.2	11	1.9	35	6.3	445	79.9	65	11.9
Inner regional	149	18.7	n.p	n.p	10	6.8	104	70.3	31	21.0
Outer regional/remote	89	11.2	n.p	n.p	n.p	n.p	54	60.7	30	33.7
Mother’s age at birth (mean (SD))	809	36.4 (1.9)	15	36.7 (1.7)	50	36.4 (1.9)	614	36.5 (1.8)	130	36.5 (1.8)
Marital status										
Partnered	645	79.9	10	1.6	39	6.1	499	77.4	97	15.0
Non-partnered	162	20.1	5	3.1	9	5.6	115	70.9	33	20.4
Smoking during pregnancy										
No	761	94.4	15	1.9	48	6.3	578	75.9	120	15.8
Yes	45	5.6	n.p	n.p	n.p	n.p	33	73.3	10	22.2
Gestational diabetes										
No	745	92.3	14	1.9	46	6.2	572	76.8	113	15.2
Yes	62	7.7	n.p	n.p	4	6.5	42	67.7	15	24.2
Mode of birth										
Non-caesarean	494	61.1	7	1.4	24	4.9	391	79.2	72	14.6
Caesarean	315	38.9	8	2.5	26	8.3	223	71.8	58	18.4
Gestational age at birth (mean (SD))	809	38.9 (1.7)	15	39.3 (1.7)	50	38.1 (2.3)	614	39.0 (1.7)	130	38.9 (1.3)
Birth weight (mean (SD))	809	3480.6 (537.8)	15	3555.4 (766.0)	50	3271.1 (656.3)	614	3497.5 (526.1)	130	3472.7 (500.1)
Child age at survey—months (mean (SD))	809	42.5 (15.6)	15	40.6 (16.8)	50	42.2 (15.1)	614	42.5 (15.5)	130	42.6 (16.7)
Child sex										
Male	437	54.0	9	2.1	32	7.3	332	75.9	64	14.7
Female	372	46.0	6	1.6	18	4.8	282	75.8	66	17.7
Average screen time per day (mean (SD))	795	1.5 (1.0)	15	1.4 (1.1)	48	1.4 (1.0)	605	1.5 (0.9)	127	1.5 (1.1)

n.p: Data not published to maintain confidentiality of small numbers.

**Table 2:** Association between the five-minute Apgar scores and neurodevelopmental delay among children aged 8−66 months.

TiFactors	Neurodevelopmental Delay					
	Gross Motor Delay		Communication Delay		Gross Motor or Communication Delay	
	AOR (95% CI)	*p*-Value	AOR (95% CI)	*p*-Value	AOR (95% CI)	*p*-Value
Five-minute Apgar score (Ref: 10)						
0−6	5.74 (1.18, 27.85)	0.03	2.89 (0.43, 19.27)	0.27	4.26 (0.95, 18.97)	0.05
7−8	4.06 (1.16, 14.12)	0.02	1.19 (0.21, 6.75)	0.83	3.51 (1.14, 10.80)	0.02
9	1.61(0.61, 4.29)	0.33	0.81 (0.28, 2.30)	0.70	1.45 (0.62, 3.38)	0.38
Mother’s country of birth (ref: Australia)						
Not-Australia	1.56 (0.57, 4.29)	0.87	1.93 (0.59, 6.34)	0.27	1.41 (0.55, 3.58)	0.46
Mother’s age at birth	0.87 (0.71, 1.05)	0.18	0.89 (0.68, 1.17)	0.41	0.88 (0.74, 1.06)	0.21
Mother’s area of residence (Ref: Major city)						
Inner regional	1.09 (0.52, 2.27)	0.23	0.87 (0.31, 2.43)	0.79	1.06 (0.54, 2.07)	0.85
Outer regional	0.83 (0.30, 2.25)	0.72	0.45 (0.10, 1.99)	0.29	0.82 (0.33, 2.02)	0.67
Marital status (Ref: Partnered)						
Non-partnered	1.23 (0.62, 2.42)	0.54	1.99 (0.89, 4.44)	0.09	1.24 (0.67, 2.29)	0.48
Smoking during pregnancy (Ref: No)						
Yes	1.03 (0.29, 3.60)	0.95	0.38 (0.04, 3.20)	0.38	1.12 (0.37, 3.35)	0.83
Gestational diabetes (Ref: No)						
Yes	1.45 (0.56, 3.71)	0.43	1.81 (0.54, 6.02)	0.33	1.37 (0.57, 3.30)	0.47
Gestational hypertension (Ref: No)						
Yes	0.33 (0.04, 2.29)	0.26	0.99 (0.20, 4.83)	0.99	0.80 (0.23, 2.77)	0.73
Mode of birth (Ref: Non-caesarean)						
Caesarean	1.22 (0.67, 2.23)	0.50	0.97 (0.43, 2.20)	0.95	1.13 (0.66, 1.95)	0.64
Gestational age at birth (weeks)	0.99 (0.80, 1.21)	0.65	0.97 (0.74, 1.26)	0.78	0.95 (0.79, 1.14)	0.93
Birth weight (grams)	0.99 (0.99, 1.00)	0.51	0.99 (0.99, 1.00)	0.61	0.99 (0.99, 1.00)	0.78
Child age at survey (months)	0.97 (0.95, 1.00)	0.07	0.98 (0.95, 1.01)	0.41	0.98 (0.95, 1.00)	0.07
Child sex (Ref: Male)						
Female	0.64 (0.35, 1.17)	0.15	0.78 (0.36, 1.70)	0.54	0.63 (0.37, 1.09)	0.10
Average screen time per day (hours)	0.96 (0.72, 1.28)	0.82	1.12 (0.80, 1.58)	0.49	0.86 (0.65, 1.13)	0.28
Child moderate to severe medical problems						
Yes	2.35 (1.22, 4.53)	0.01	5.51 (2.55, 11.91)	<0.001	2.76 (1.51, 5.02)	<0.001

AOR, adjusted odds ratio; CI, confidence interval.

## Data Availability

Data are available from the Australian Longitudinal Study on Women’s Health. Information about data access is made available at https://alswh.org.au/.

## Supplementary Materials

The following are available online at https://www.mdpi.com/article/10.3390/ijerph18126450/s1, Table S1: Association between the five-minute Apgar scores (continuous) and neurodevelopmental delay among children aged 8−66 months.

## Queensland (QLD) Health

including the Statistical Services Branch for the Qld Perinatal Data Collections.

Department of Health Western Australia (WA), including the Data Linkage Branch, and the WA Midwives Notification System

South Australia (SA) Northern Territory (NT) Datalink and SA Department for Health and Wellbeing and NT Department of Health for the SA Perinatal Statistics and NT Perinatal Trends Data Collections

Tasmanian Data Linkage Unit and the Department of Health Tasmania for the Perinatal Data Collections.

The Department of Health and Human Services Victoria, Centre for Victorian Data Linkage, for Victorian Perinatal Data Collection. We are grateful to the Consultative Council on Obstetric and Paediatric Mortality and Morbidity (CCOPMM) for providing access to the data used for this project and for the assistance of the staff at Safer Care Victoria. The conclusions, findings, opinions, and views or recommendations expressed in this paper are strictly those of the author(s). They do not necessarily reflect those of CCOPMM.
