# Supplementary material for: The Association between the Five-Minute Apgar Score and Neurodevelopmental Outcomes among Children Aged 8−66 Months in Australia
Source: Int J Environ Res Public Health. 2021 Jun 15;18(12):6450. doi: 10.3390/ijerph18126450 (PMC8296269; doi:10.3390/ijerph18126450)
Supplement: Supplementary file 1 [file ijerph-18-06450-s001.zip › ijerph-1244870-supplementary.pdf]

Table S1: Association between the five-minute Apgar scores (continuous) and neurodevelopmental delay among children aged 8−66 months

**Factors**	**Neurodevelopmental delay**					
	**Gross motor delay**		**Communication delay**		**Gross motor or communication delay**	
	**AOR (95% CI)**	**P-Value**	**AOR (95% CI)**	**P-Value**	**AOR (95% CI)**	**P-Value**
Five minute Apgar Score	0.72 (0.54, 0.90)	0.005	0.73 (0.55,0.97)	0.03	0.74 (0.60, 0.93)	0.01
Mother's country of birth (ref: Australia)						
Not-Australia	1.50 (0.54, 4.14)	0.42	1.75 (0.53, 5.72)	0.35	1.29 (0.51, 3.29)	0.58
Mother's age at birth	0.85 (0.69, 1.04)	0.13	0.88 (0.67, 1.15)	0.37	0.88 (0.73, 1.05)	0.17
Mother's area of residence (Ref: Major city)						
Inner regional	1.05 (0.50, 2.19)	0.88	0.87 (0.31, 2.44)	0.80	1.02 (0.52, 1.99)	0.94
Outer regional	0.81 (0.30, 2.18)	0.69	0.49 (0.11,2.14)	0.29	0.80 (0.33, 1.95)	0.63
Marital status (Ref: Partnered)						
Non-partnered	1.23 (0.62, 2.41)	0.54	2.13 (0.96, 4.75)	0.06	1.24 (0.67, 2.28)	0.48
Smoking during pregnancy (Ref: No)						
Yes	1.00 (0.28, 3.46)	0.99	0.41 (0.05, 3.43)	0.41	1.11 (0.37, 3.30)	0.84
Gestational diabetes (Ref: No)						
Yes	1.43 (0.56, 3.67)	0.44	1.82 (0.54, 6.09)	0.32	1.37 (0.57, 3.29)	0.47
Gestational hypertension (Ref: No)						
Yes	0.32 (0.04, 2.25)	0.25	1.02 (0.21, 4.99)	0.97	0.79 (0.23, 2.75)	0.72
Mode of birth (Ref: Non-caesarean)						
Caesarean	1.27 (0.70, 2.31)	0.42	0.97 (0.43, 2.19)	0.95	1.15 (0.67, 1.98)	0.58
Gestational age at birth (weeks)	1.03 (0.83, 1.28)	0.75	1.03 (0.76, 1.39)	0.82	0.97 (0.80, 1.18)	0.82
Birth weight (grams)	0.99 (0.99, 1.00)	0.42	0.99 (0.99, 1.00)	0.60	0.99 (0.99, 1.00)	0.69
Child age at survey (months)	0.97 (0.95, 1.00)	0.06	0.98 (0.95, 1.01)	0.35	0.97 (0.95, 1.00)	0.05
Child sex (Ref: Male)						
Female	0.62 (0.34, 1.14)	0.12	0.80 (0.37, 1.73)	0.58	0.62 (0.36, 1.08)	0.09
Average screen time per day (hours)	0.96 (0.72, 1.28)	0.78	1.13 (0.80, 1.59)	0.66	0.87 (0.66, 1.14)	0.32
Child moderate to severe medical problems						
Yes	2.28 (1.16, 4.49)	0.01	5.59 (2.59, 12.06)	<0.001	2.71 (1.49, 4.93)	0.01

AOR: Adjusted odds ratio; CI: Confidence interval
